# Volume XXVIII

**Published:** 1888-08

**Authors:** Thos. Lothrop


### Editorial.

### *VOLUME XXVIII.*

The present number of the JOURNAL, the initial number of the forthcoming volume, is presented to our readers in a new and improved dress, and with the addition to its editorial corps of Dr. William Warren Potter, of this city, who is so well known to the profession as an accomplished physician and a vigorous and graceful writer, that he needs no other introduction.

In 1879, the JOURNAL was transferred, by purchase, from the late Prof. Julius F. Miner to a corps of five editors, viz.: Drs. Davidson, Mynter, Van Peyma, Howe, and the writer. Of this number, Drs. Mynter, Van Peyma and Howe retired, while the death of the lamented Davidson placed the entire responsibility of its publication with the surviving editor.

For several years we depended, in the professional and scientific departments of this work, upon the able and judicious supervision of the deceased editor, to whose earnest labors in its behalf we acknowledge with gratitude that the excellent reputation of the JOURNAL is largely due. We beg also to give due praise to the associate editors, who have ably assisted in the preparation of the monthly numbers. It may be proper to state that, for two or three years, the writer has only occasionally wielded the editorial pen, and then only when it became necessary to speak on subjects of vital interest to the profession in language so positive that it could not be misunderstood. It is his purpose, in the present volume, to resume the work laid aside on account of urgent professional duties, and to take his full share of the responsibility of the management of the JOURNAL.

We think we voice the sentiments of the profession when we state that, in the selection of our new editorial confrere, we have consulted the interests of the JOURNAL and of our numerous readers and subscribers. Our pages, in previous numbers, have contained able and interesting articles from his pen, an earnest of his efforts in his new relation, now that he assumes an equal responsibility in the financial and editorial work.

We may refer most hopefully to the future of the JOURNAL under its new management. It is intended to furnish our readers with the best thought on all medical subjects, and to keep the JOURNAL abreast with the scientific progress of the age. We may here state that, as a reflex of medical thought, its monthly numbers will measure the activity of the profession in this portion of the state. Upon their earnest labors we depend for aid and encouragement in our work.

The JOURNAL will not be the organ of any school or clique. It will be strong in its position, independent in its convictions, and fearless in its expression of opinions on all questions affecting the interests of the profession. We aim at no popularity purchased at the expense of duty and of right. If we criticize boldly, it will be for the reason that, as medical journalists, the profession, in its high and noble aims, demands the independent attitude we assume.

On the important subject now uppermost in the mind of the medical profession throughout the country, that of higher medical education, the JOURNAL will continue in the future to be the champion of such an advance in the curriculum of our schools, and of the preliminary education of matriculants, that the profession of medicine may not receive the merited criticism of the learned professions on account of the ignorance and illiteracy of its members.

We beg to express to our readers and subscribers our deep sense of obligation for the support and patronage given in the past, and we confidently hope that, by an earnest effort to improve the JOURNAL, we may deserve an increased favor in the future.

THOS. LOTHROP.

